# Accumulation, Source Identification, and Cancer Risk Assessment of Polycyclic Aromatic Hydrocarbons (PAHs) in Different Jordanian Vegetables

**DOI:** 10.3390/toxics10110643

**Published:** 2022-10-27

**Authors:** Farh Al-Nasir, Tahani J. Hijazin, Mutaz M. Al-Alawi, Anwar Jiries, Osama Y. Al-Madanat, Amal Mayyas, Saddam A. Al-Dalain, Rasha Al-Dmour, Abdalrahim Alahmad, Mufeed I. Batarseh

**Affiliations:** 1 Faculty of Agriculture, Mutah University, Karak 61710, Jordan; 2 Biology Department, Faculty of Science, Mutah University, Karak 61710, Jordan; 3 Ministry of Environment, Amman 11941, Jordan; 4 Chemistry Department, Faculty of Science, Mutah University, Karak 61710, Jordan; 5 Prince Faisal Center for the Dead Sea, Environmental and Energy Research, Mutah 61710, Jordan; 6 Department of Pharmacy, Faculty of Health Science, American University of Madaba, Amman 11821, Jordan; 7 Al-Shoubak University College, Al-Balqa Applied University, Al-Salt 19117, Jordan; 8 Institut für Technische Chemie, Leibniz Universität Hannover, 30167 Hannover, Germany; 9 Academic Support Department, Abu Dhabi Polytechnic, Abu Dhabi P.O. Box 111499, United Arab Emirates

**Keywords:** vegetables, soil, water, PAHs, cancer risk, source identification, bioconcentration, diagnostic ratio

## Abstract

The accumulation of polyaromatic hydrocarbons in plants is considered one of the most serious threats faced by mankind because of their persistence in the environment and their carcinogenic and teratogenic effect on human health. The concentrations of sixteen priority polycyclic aromatic hydrocarbons (16 PAHs) were determined in four types of edible vegetables (tomatoes, zucchini, eggplants, and cucumbers), irrigation water, and agriculture soil, where samples were collected from the Jordan Valley, Jordan. The mean total concentration of 16 PAHs (∑16PAHs) ranged from 10.649 to 21.774 µg kg^−1^ in vegetables, 28.72 µg kg^−1^ in soil, and 0.218 µg L^−1^ in the water samples. The tomato samples posed the highest ∑16PAH concentration level in the vegetables, whereas the zucchini samples had the lowest. Generally, the PAHs with a high molecular weight and four or more benzene rings prevailed among the studied samples. The diagnostic ratios and the principal component analysis (PCA) revealed that the PAH contamination sources in soil and vegetables mainly originated from a pyrogenic origin, traffic emission sources, and biomass combustion. The bioconcentration factors (BCF) for ∑16PAHs have been observed in the order of tomatoes > cucumbers and eggplants > zucchini. A potential cancer risk related to lifetime consumption was revealed based on calculating the incremental lifetime cancer risk of PAHs (ILCR). Therefore, sustainable agricultural practices and avoiding biomass combusting would greatly help in minimizing the potential health risk from dietary exposure to PAHs.

## 1. Introduction

Contamination by polycyclic aromatic hydrocarbons (PAHs) in foods gained great environmental concern from researchers all over the world. In addition, it has become a food safety matter in many different countries because of their ubiquitous presence in environmental compartments (air, water, plant, and soil ecosystems) and their well-recognized carcinogenicity, toxicity, mutagenicity, persistence, and mobility throughout the environment [1,2].

PAHs can be classified into two groups: (i) low molecular weight (LMW) PAHs, which contain up to two or three aromatic cycles and (ii) high molecular weight (HMW) PAHs, which contain at least four to six aromatic rings [1,2]. HMW-PAHs can exit in both vapor and particulate phases and are more stable in the environment due to their low volatility, resistance to leaching as well as the ability to biodegrade, while LMW-PAHs normally exist in the vapor phase and temporarily remain in the environment because of their high water solubility, volatility, and lower lipophilicity [1,2].

PAHs are widely detected at various levels in soil [3], water [4], aerosols [5], sewage sludges [6], marine organisms [7], and plants [8,9]. Several methods have been proposed for the removal of organic pollutants such as PAHs from the environment [10,11,12,13,14,15]; however, most of them possess several disadvantages. The contamination of food with PAHs has received great environmental concern due to their carcinogenicity, toxicity, mutagenicity, persistence, and mobility throughout the environment [1,2].

As PAHs are lipophilic and semivolatile compounds, they can be accumulated by vegetables. However, this depends on the physicochemical characteristics of the individual PAH compounds such as water solubility, volatility, and lipophilicity [16]. PAHs are strongly adsorbed onto the organic part of soils, where approximately 90% of total PAHs remain on surface soils for long periods [17,18]. The main routes involved in the migration of PAHs from soil to plant tissues are by the uptake by roots and sorption from soil particles [19]. The contents of PAHs in vegetables increase with the increasing PAH content in the soil [20]. In general, the PAH uptake by vegetables and fruits primarily occurs via four types of routes including contaminated soil uptake, contaminated water uptake, airborne particulates deposition (from the air), and autogenous biosynthesis of PAHs in tissues [21]. Food chain contamination with PAHs has an adverse impact on human health as around 30% of human cancers have been always associated with low exposure to PAHs in the diet [22].

PAH contamination in vegetables has been studied worldwide [23,24,25,26,27]. Variations in PAH accumulation appear to be noticed in varieties of the same vegetable [28]. Among individual PAH congeners, the most abundant PAH compounds in vegetables were fluorene, phenanthrene, anthracene, fluoranthene, and pyrene [26,29]. A variation in PAH concentration was reported for different parts of the plants, where root vegetables showed higher values than stem vegetables, and this is ascribed to the difference in their growth structures [23]. On the other hand, plant morphology plays a role in PAH concentration as the greater the surface area of the leaves the higher PAH concentration shown due to the increasing PAH absorption from the atmosphere [30,31,32]. Tuteja et al. reported that leafy vegetables such as spinach, white gourds, fenugreeks, chilis, ribbed gourds, and cauliflowers were more contaminated with PAHs compared to underground ones such as potatoes, radishes, and turnips [32]. Vegetables with waxy surfaces can accumulate particle-bound HMW-PAHs via atmospheric deposition and LMW-PAHs via surface adsorption [33]. At the same time, however, it has been observed that vegetables with a large and ragged surface such as cauliflowers, cabbages, and grapes enable the trapping of particulate matter which results in an increased contribution of four-ring PAHs [34]. Li et al. studied the presence of 16 PAHs in six commonly consumed vegetables (Napa cabbages, beans, cucumbers, tomatoes, Chinese leeks, and celery) collected from the markets in Shandong, China. They found that they were all polluted with PAHs at an alarming level, of which celery contained the highest ∑16PAH (413.2 µg kg^−1^), while cucumbers contained the lowest ∑16PAH (14.2 µg kg^−1^) [26].

As vegetables are considered one of the fundamental parts of the Jordanian daily diet, it is essential to provide periodic information regarding the accumulation of PAHs in the agricultural soils, irrigation water, and vegetables for the local authorities and consumers. Batarseh (2011) investigated the concentration levels of 16 PAHs in sewage sludge samples in the As-Samra area in Jordan which varied from 35 μg g^−1^ to 70 μg g^−1^ [35]. However, there is a shortage of documented reports [27] regarding the accumulation of PAHs in the study area and in its edible plant products, especially vegetables. Thus, the focus of this study is to investigate PAH contamination in one of the most famous agricultural spots in Jordan. Specifically, our study had the following objectives: (1) to evaluate the concentrations of 16 priority PAHs in agricultural soils and edible parts of four types of vegetables (cucumbers, eggplants, zucchini, and tomatoes); (2) to identify the probable sources of PAHs by using PAH diagnostic ratios and PCA; (3) to undertake an assessment of the PAH bioconcentration in these vegetables; and (4) to calculate the ILCR posed through vegetable consumption. The obtained results may be greatly helpful in promoting sustainable agricultural practices and minimizing the risk of human exposure to contamination by emerging pollutants.

## 2. Materials and Methods

### 2.1. Chemicals and Reagents

The solid phase extraction SPE cartridges OASIS HLB (6 mL/500 mg) were obtained from Waters^®^, Ireland. Standard mixture of the 16 PAHs dissolved in acetonitrile at 10 µg L^−1^ was procured from Merck (Darmstadt, Germany). It consisted of the following PAHs, namely: naphthalene (NAP), acenaphthylene (ACY), acenaphthene (ACE), chrysene (CHR), anthracene (ANT), fluorene (FLO), fluoranthene (FLA), phenanthrene (PHE), pyrene (PYR), benzo[b]fluoranthene (BbF), benzo[k]fluoranthene (BkF), indo [1,2,3,c,d]pyrene (IcdP), benzo[a]anthracene (BaA), benzo[g,h,i]perylene (BghiP), benzo(a)pyrene (BaP), and dibenzo[a,h]anthracene (DahA). Milli-Q ultrapure water with electrical conductivity of 18.2 MΩ cm was obtained using a water purification system from Waters, Milford, USA. The stock solutions of 50 µg µL^−1^ were prepared in acetonitrile for the individual PAHs, while a mixture of the 16 PAHs was prepared on weekly basis in methanol at different concentrations ranging from 50 ng µL^−^^1^ to 1000 ng µL^−1^. The standard calibration solutions were prepared on daily basis using a mixture solution 50:50 (*v*/*v*) of water and methanol (0.1% formic acid). Solvents were either of analytical or HPLC grade (Sigma-Aldrich, St. Louis, MO, USA).

### 2.2. Study Area

**Figure 1:**
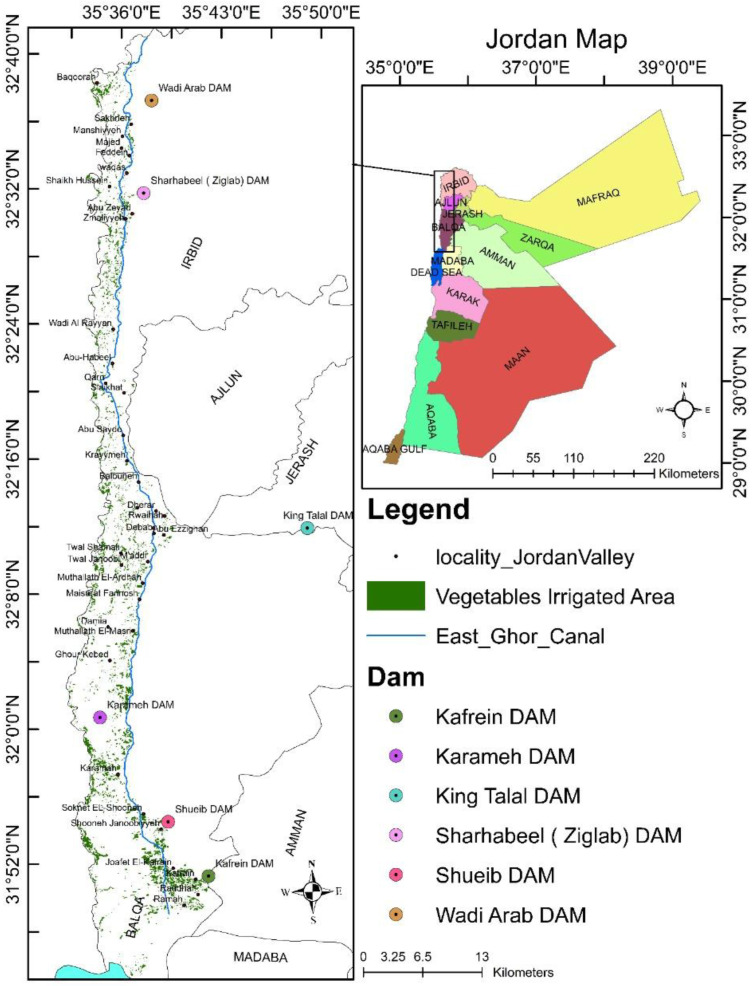
Geographical location of the study area.

The study area was located close to Deir Alla village in the middle of the Jordan Valley, Jordan(Figure 1). It extends from Qarn in the north to Southern Shooneh city in the south which is positioned between the Jordan River and East Ghor Canal (King Abdullah Canal), as shown in Figure 1. The irrigation water comes from the King Abdullah Canal (KAC) and King Talal Dam (KTD). The water quantity and quality may vary seasonally along the waterway depending on the water resources [36], where water quality of King Abdullah Canal is considered cleaner than that of the King Talal Dam [36].

This study area is categorized by its mild winters and hot summers with annual precipitations of around 300 mm [37]. The common cultivated vegetables in the area are cucumbers, eggplants, tomatoes, and zucchini, and the soil type is a calcareous soil [38]. The investigated site can be characterized by a medium traffic density that has minimal contamination from the adjacent environment [39].

### 2.3. Sampling

Soil, water, and vegetable samples were collected from the same area at the end of the growing season that extended from November to December 2018. Four types of ripe vegetables were included in the study: cucumbers, eggplants, tomatoes, and zucchini. Sampling activity was accomplished based on the standard sampling procedure as per the European Commission (2002) [40].

Ten samples per product, in addition to subsamples, were collected from various locations throughout the investigation site. Each 1 kg sample was cleaned with distilled water, dried with paper towels, placed in a sterile bag, and stored at 4 °C before the laboratory tests.

A total of six irrigation water samples were collected during the year 2018. All the samples were collected in the same manner from the canals connected to the farms. Triplicate samples were collected by gathering the run water inside a 1 L amber glass bottle, acidified to pH 2 using 1M solution of hydrochloric acid (HCl), and stored at 4 °C prior to analysis.

Furthermore, twenty soil samples were obtained using a stainless-steel auger at two depths: the lower zone (15–30 cm) and the upper surface zone (0–15 cm). Then the samples from the two depths were mixed to avoid the remarkable differences in the concentration of the LMW-PAHs in both depths due to their evaporation from the above (upper) soil parts. Each homogeneous sample was composed of five subsamples (one from the center and four from the corners of the area); it covered a plot of 100-m × 100-m, and subsamples were thoroughly mixed to form approximately 500–1000 g. The soil samples were stored in a sterile dark container at 2 °C prior to extraction procedure. As PAHs are sensitive to light and to avoid degradation or contamination, samples of all kinds including soil, plant, and water were extracted within three days.

### 2.4. Sample Preparation and PAH Extraction

#### 2.4.1. Sample Pretreatment

Vegetable samples were rinsed using Milli-Q ultrapure water, and edible peels were removed to assess PAH concentration. Samples were homogenized and prepared using a high-speed blender, and the smooth purée of the vegetable was kept at −20 °C until the analysis was performed.

The triplicated water samples were mixed, and one liter was filtrated using a vacuum filtration unit supplied with a membrane pare of 35–40 μm pore size obtained from Hahnemühle GmbH (Dassel, Germany). The filtered water sample was transferred to a SPE system obtained from Agilent Varian, Santa Clara (CA, USA) and equipped with an Oasis HLB cartridge and a vacuum pump from Waters Corporation (Milford, MA, USA). Meanwhile, soil samples were homogenized by sieving (<2 mm), and then stored in a solvent-cleaned glass jar at −20 °C until the performance of the instrumental analysis [27].

#### 2.4.2. Extraction of PAHs

Water sample was passed through a preconditioned SPE cartridge at a flow rate of 1 mL m^−1^ and washed in sequence with 6 mL of ethyl acetate, 6 mL of methanol, and 6 mL of acidified HPLC water. The SPE cartridge was loaded at a flow rate of 3 mL min^−1^ and washed out with 6 mL 5% methanol in water. The retained compounds were eluted using 2 mL of ethyl acetate at a flow rate of 1 mL min^−1^, and the eluent was passed through an anhydrous sodium sulfate column (2 g) to remove any excess water. Finally, the eluent was concentrated to 0.5 mL using a gentle stream of nitrogen and stored in an amber glass vial.

Meanwhile, soil and vegetable samples were prepared using a solid liquid extraction technique explained by Al Nasir and Batarseh (2008) [27]. Briefly, 50 g of soil or 100 g of vegetables wet homogenized samples was placed into a 500 mL Erlenmeyer flask. Samples were placed on a horizontal shaker overnight after adding 100 mL of acetone for the vegetables and 100 mL of a 2:1 acetone/water (*v*/*v*) mixture for the soil. The liquid–liquid partitioning was done by 1 h of shacking with 15 g NaCl and 100 mL cyclohexane. The organic layer of the analyte was placed into a 250 mL Erlenmeyer flask and dried using anhydrous sodium sulfate (15 g). The extract was concentrated to 1 mL using the rotary evaporator and then dried using a gentle stream of N_2_ gas. The residue was dissolved in 3 mL hexane and then dried again and redissolved in 5 mL of a mixture of 1:1 (*v*:*v*) ethyl acetate and cyclohexane. Finally, the cleanup was performed using a microfiltration syringe made of a polytetrafluoroethylene (PTFE). The filtrate was concentrated to 1 mL and passed through a deactivated alumina column (10 g) loaded with 2 g of anhydrous sodium sulfate to the top of the column. The sample was eluted with 50 mL of n-hexane and reconcentrated to 4 mL with a help of a gentle nitrogen stream. Finally, the elute was divided into four portions of 1 mL vials and kept at −20 °C for further HPLC analysis.

### 2.5. Instrumental Analysis

A system of HPLC model Agilent 1200 supplied with a symmetric biphenyl column (4.6 mm × 250 mm, 5.0 μm diameter), diode array model DE64262847, and a fluorescence detector model DE90959767 were used for the analysis of the 16 PAHs. The separation technique was adjusted using a mobile phase composed of Milli-Q ultrapure water (A) and acetonitrile (B), while a gradient elution method was optimized at a flow rate of 1 mL min^−1^ using the following protocol: 0–10 min, 40% A; 10–35 min, 10% A; 35–40 min, 0% A; 40–40.1 min, 40% A; and kept constant to 45 min. The sample injection volume was 20 µL, and the column oven temperature was kept constant at 35 °C. A blank sample was used to spot any contamination during the treatment process. The fluorescence detector was designed to switch its excitation and emission wavelengths at different frequencies according to the following values: an excitation wavelength of 260 and 260 nm, and emission wavelengths of 352, 420, and 460 nm. Finally, the 16 PAH concentrations were determined according to US-EPA priority based on internal standards.

### 2.6. Quality Assurance and Quality Control

The analytical method was validated using blank, standard spiked recoveries, limit of detection (LOD), and limit of quantification (LOQ). The resulting peak area, peak height, and retention time were statistically evaluated for both LOD and LOQ based on signal-to-noise ratio (S/N) of 3 and 10, respectively [41,42]. Each compound peak identification and quantitation was performed using a single standard solution, and the identification was made by performing a relative retention time technique. The analyte quantification was accomplished using an external calibration method by comparing the peak areas of the samples to those of the standard solution. The LOD and LOQ of PAHs were calculated using a statistical method that considered the concentration of analyte in the sample.

**Figure 2:**
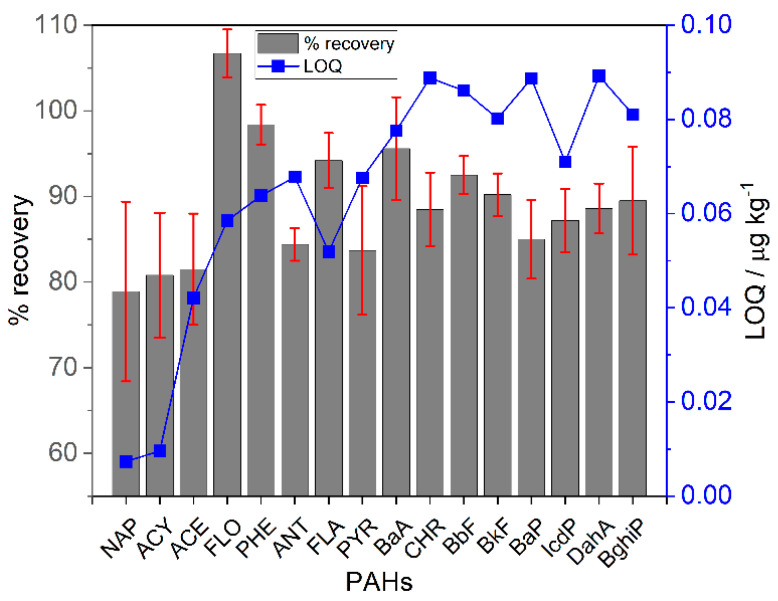
% recovery and LOQ of the soil samples.

The calculated LOD and LOQ ranges were 3.10 to 27.03 ng kg^−1^ and 7.35 to 89.20 ng kg^−1^ for the soil and plant samples (Figure 2), respectively, while LOD and LOQ ranged from 0.050 to 3.96 ng L^−1^ and 0.17 to 13.15 ng L^−1^ for the water samples, respectively. The precision and linearity of analytical procedures were evaluated using the relative standard deviation (RSD) ± 20% and an accepted standard for calibration R^2^ > 0.995 [42], while the variation coefficients in the concentrations of different PAHs between the duplicate samples were <10%.

Furthermore, recovery studies were performed using a blank sample and a 50 μg kg^−1^ concentration level of each PAH spiked with different samples of soil, vegetables, and 10 μg L^−1^ for the water samples. The mean recovery rate of these compounds was between 79 to 107%, with RSD values ≤10. Figure 2 shows the % recovery of the soil samples. The analytical results of the studies performed on the different compounds agreed with the recovery rates reported in previous studies [35,39,43,44].

### 2.7. Estimates of Cancer Risk from PAHs

The cancer risk estimation was calculated using the incremental lifetime cancer risk (ILCR) which can be defined as the incremental probability of a person having cancer during a lifetime via different exposure pathways to a potential carcinogen, such as PAHs [1]. The toxicity equivalent factor (TEF) is a component of the comparative potency approach, and it is broadly used for cancer risk assessment of PAHs in humans via consuming contaminated vegetables [26]. The ILCR was calculated according to the relative Equation (1) [24,25]:

(1) $$\mathrm{ILCR}=\frac{{\mathrm{TEQ}}_{\mathrm{BaP}}\times {\mathrm{IR}}_{i}\times \mathrm{EF}\times \mathrm{ED}\times \mathrm{SF}\times \mathrm{CF}}{\mathrm{AT}\times \mathrm{BW}}$$

where ILCR is the incremental lifetime cancer risk of dietary exposure, BW is the body weight for an adult (70 kg), CF is a conversion factor (10^−6^ mg ng^−1^), ED is the exposure duration (70 years), SF is the oral cancer slope factor of benz[a]pyrene (7.3 (mg kg^−1^ day^−1^)^−1^) [45], EF is the exposure frequency (365 d yr^−1^), AT is averaging time (days) for cancer genic development [AT = ED × 365] (i.e., 25,550 days) [24], and IR_i_ is the amount of vegetables, ingested per day (56.16 g day^−1^ for tomatoes, 39.18 g day^−1^ for cucumbers, 12.33 g day^−1^ for eggplants, and 9.86 g day^−1^ for zucchini) [37].

Total toxic BaP equivalent (TEQ_Bap_) for PAHs was calculated by the following Equation (2)

(2) $${\mathrm{TEQ}}_{\mathrm{BaP}}=\sum_{i=1}^{n}C_{i}\times {\mathrm{TEF}}_{i}$$

where C_i_ is the concentration of the individual PAH in vegetables, and TEF_i_ is the corresponding toxic equivalency factor for PAHs. In this method, the TEF of 16 PAH congeners are as follows: BghiP = 0.01, ACY =0.001, ACE = 0.001, PYR = 0.001, PHE = 0.001, CHR = 0.01, FLA = 0.001, FLO = 0.001, BaA = 0.1, ANT = 0.01, BbF = 0.1, BkF = 0.1, BaP = 1, DahA = 1, IcdP = 0.1, and NAP =0.001 [46]. For statistical reasons, the concentration of PAH species that was lower than the detection limit was supposed to be zero.

### 2.8. Data Analysis

A principle components analysis (PCA) was employed using SPSS 26.0 software to reduce a group of original variables of the measured PAHs in the soil samples and extract a number of principal components (PCs) for analyzing the relationships among the observed variables. Kaiser’s Rule was used to study the number of factors extracted from the variables and deduced by a screen test. This criterion keeps only factors with eigenvalues >1. PCA was accomplished on concentration data from 15 PAHs, while PHE was excluded because of its low detection frequencies in most of the soil samples.

## 3. Results and Discussion

### 3.1. PAHs in Irrigation Water

**Figure 3:**
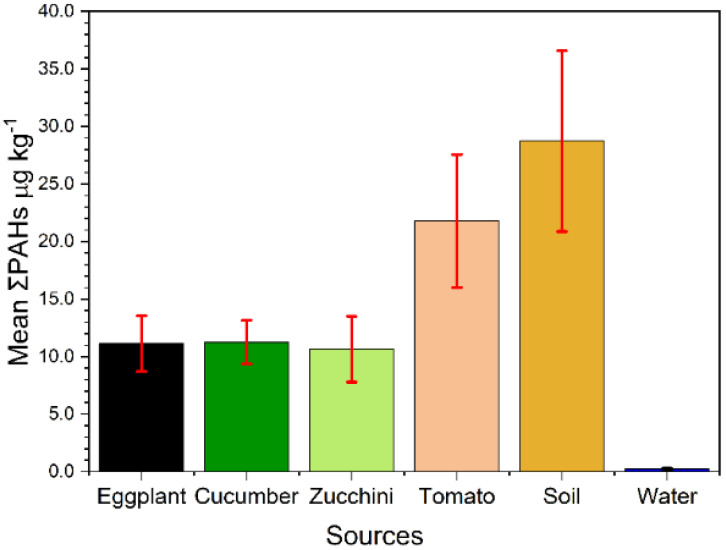
Mean ∑16PAHs in each type of plant, soil, and water samples from the studied area.

The concentrations of the detected 16 PAHs detected in the irrigation water of the investigated fields are summarized in Table 1. The HMW-PAHs were completely dominant in the water, while the LMW could not be detected. The dominant PAHs in the irrigation water samples (BaA and BaP) were detected in a concentration of 0.066 and 0.097 µg L^−1^ in about 71 and 100% of the analyzed samples, respectively. Although they were found at low concentrations, these two detected PAHs (BaA and BaP) are known for their high risk of cancer. Therefore, the irrigation water could have a significant influence on concentrating these two PAHs in the investigated vegetables; as shown in Table 1, they were detected in high concentrations in all of the analyzed samples. Figure 3 shows that the lowest total mean concentration of PAHs was found in the water samples. This can be attributed to the lower solubility of these compounds in water. In addition, the total concentration of the detected PAHs in the water was 0.211 µg kg^−1^. This concentration is higher than the PAH content of the irrigation water taken from the King Abdullah Canal/Jordan Valley [39]. This can be attributed to the mixing of the irrigation water in this study from different resources, i.e., King Abdullah Canal and King Talal Dam, where the water quality in the King Abdullah Canal is considered to be cleaner than that from the King Talal Dam [36].

### 3.2. PAHs in Soil

The concentrations of the analyzed PAHs in the soil are shown in Table 1. Our results in Figure 3 show that the mean ∑16PAHs in the soil is much higher at about 28.722 µg kg^−1^ than the other vegetables and water samples. This may be due to the continuous accumulation of these PAHs during irrigation as well as a longer exposure to the ambient environment than the vegetables. The PAH pattern in the soil is quite different from those of the water and vegetables, where in the soil the LMW-PAHs were higher with a concentration of 15.730 µg kg^−1^ in comparison to the HMW-PAH concentration of 12.992 µg kg^−1^. The carcinogenic indicators of BaP, ANT, and ACE in the soil comprise the higher contents of the total PAHs in concentrations of 6.776, 6.269, and 5.450 µg kg^−1^, respectively.

Although the investigated area is characterized by its hot climate, the concentration of LMW is higher than that of the HMW-PAHs in the soil. This could be ascribed to a recent and continuous input of these compounds by frequent irrigation. However, the dominant types were BaA and BaP in all of the soil samples with very high concentrations due to their high stability and toxicity compared to the LMW-PAHs.

To determine the level of toxicity by PAHs in the soil, a classification defined by Maliszewska–Kordybach [47] was utilized. This classification relies on the evaluation of the ∑16PAH level of soil contamination by PAHs. The results show that the studied area can be considered as “not contaminated soil” as the ∑16PAHs ≤200 µg kg^−1^.

Additionally, the Canadian Council of Ministers of the Environment proposed another classification containing three major classes, specifically, class A, or clean soil when the concentration of BaP is less than 100 µg kg^−1^; class B, or slightly polluted soil when the concentration of BaP is less than 1000 µg kg^−1^ (further investigation is required); and class C, or seriously polluted soil when the concentration of BaP is up to 10,000 µg kg^−1^ (immediate remediation is needed) [48]. Based on this classification, the investigated agricultural soils was clean soil.

In order to evaluate the quality of the soil and the level of contamination in the studied area, the level of the PAH concentrations in different areas was compared with this study. The concentration of the PAHs in the studied area in Jordan (28.722 µg kg^−1^) was significantly lower than those in Delhi, India (1910 µg kg^−1^), the Pearl River Delta, China (1503 µg kg^−1^), and Japan (320 µg kg^−1^) [49,50] but slightly higher than the levels registered in some places such as Henan, China (24.40 µg kg^−1^) and Canada (16 µg kg^−1^) [51,52]. Based on these comparisons, the studied agricultural soil near Jordan was slightly contaminated with PAHs compared to the soils in other parts of the world.

The ability of the compound to be taken up by the plants through the roots is governed by its physiochemical properties, such as hydrophobicity, which can be deduced from the logP (decadal logarithm of K_ow_) of the individual compound.

### 3.3. PAHs in Vegetables

The concentrations of the priority 16 PAHs were determined in four different types of vegetables (eggplants, tomatoes, zucchini, and cucumbers) and are shown in Table 1. The total PAHs (∑16PAHs) showed the highest concentration (21.774 µg kg^−1^) in tomatoes and the lowest one in zucchini (10.649 µg kg^−1^), as shown in Figure 3.

In general, high molecular weight PAHs, especially BaP and BaA ranging from 7.844 to 12.424 µg kg^−1^, showed higher concentrations than the low molecular weight PAHs which ranged from 2.751 to 9.350 µg kg^−1^. It has been shown that three-ring PAHs dominated in cucumbers, tomatoes, and apples, i.e., plants with a smooth waxy surface [34].

In addition, the concentration of several PAHs was below the detection limit in many samples. Comparing our results with other investigations, it was found that the total PAHs were much higher in this study compared with Egyptian vegetables [29], but it was close to Chinese vegetables of the same type [53], which can be attributed to the surrounding environment rather than physiological reasons, where Zhang et al. analyzed many farmland soils near highways and found a high PAHs contamination in the soils due to vehicle exhausts [54]. While, in other reports, the higher ∑PAH content was found in tomatoes (9.50 μg kg^−1^) among several types of vegetables [55], which is lower than our finding.

It is worth mentioning that the difference in the pattern of the detected PAHs in the different vegetable types is due to species–specific characteristics, not the soil or the environmental conditions, as all of the sampling sites are of similar soil and irrigated with the same type of water. Tomatoes showed the highest total PAH concentration, whereas ANT, BaP, and BaA constituted the highest concentration in nearly all of the samples (100%), with concentrations of 6.119 µg kg^−1^, 5.436 µg kg^−1^, and 3.718 µg kg^−1^, respectively. However, fluorine was not detected in any of the tomato samples.

For eggplants, all of the PAHs were detected in all of the samples except BkF and DahA. BaA was predominant in all the samples (100%) at a concentration of 3.236 µg kg^−1^. ACY, ACE, FLO, PHE, and BkF could not be detected in all of the zucchini samples. However, BaP and BaA showed the highest concentrations in all of the samples with a mean concentration of 3.333 µg kg^−1^ and 2.641 µg kg^−1^ for eggplants, respectively. In cucumbers, BaP (3.471 µg kg^−1^) and BaA (2.807 µg kg^−1^) were the prevalent PAHs, while ACY, PHE, CHR, and BkF could not be detected in all of the samples.

Benzo(a)pyrene, which is widely used as an indicator for carcinogenic PAHs, was found in significant concentrations in all of the samples (100%) of the four studied vegetables, ranging from 3.234 µg kg^−1^ in eggplants to 5.4361 µg kg^−1^ in tomatoes.

In most of the reported studies, LMW-PAHs are more prevalent or dominant in the studied samples than the HMW-PAHs in many studied vegetable species [26,56]. However, in our study, HMW-PAHs are predominant in the analyzed samples which may be ascribed to reasons that will be discussed in Section 3.4.

The high concentration of PAHs in tomatoes related to other analyzed species can be attributed to their growth period and irrigation requirements. Tomatoes, with the highest growth period, were found to have the highest LMW-PAHS which can be attributed to a higher consumption of irrigation water which is known to be predominant with LMWPAHs.

The variations in PAH concentrations between tomatoes and other analyzed plants might be attributed to the water consumption and growth period of these plants; tomatoes have the highest water consumption of 400–600 mL compared to other investigated vegetable species uptake and store higher amounts of LMWPAHs in their fruits. The PAH profile revealed a higher amount of detected HMW-PAHs compared to LMW-PAHs with a ratio of 0.753 (LMW-PAHs/HMW-PAHs) in tomatoes compared to 0.329–0.371 in other analyzed plant samples. This means a two-fold higher solubility of LMW-PAHs in tomatoes related to other vegetable species. Our results are in good agreement with the local study by Al-Nasir and Batarseh (2008) [27]

### 3.4. Transfer of PAHs

These PAHs can transfer into plants either through the air by the deposition of particles present in the air resulting from traffic emissions onto the external epidermis of the vegetables or through the soil to the root, followed by their translocation to the aerial parts [28]. The ability of the compound to be taken up by the plants through the roots is governed by its physiochemical properties, such as hydrophobicity, which can be deduced from the octanol/ water partition coefficient or log *p* (decadal logarithm of Kow) of a certain compound. The uptake into the plants is decreased with the increase in the Log *p*, where substances that have a log *p* value between −1.0 to 4.0 are able to passively diffuse into the plant roots and then be translocated into the shoots [57]. Thus, the LMW-PAHs are more easily taken up and accumulated into the plants than the HMW-PAHs due to their relatively low Log *p* values, which make them more stable in the soil and less volatile and degradable [58].

**Figure 4:**
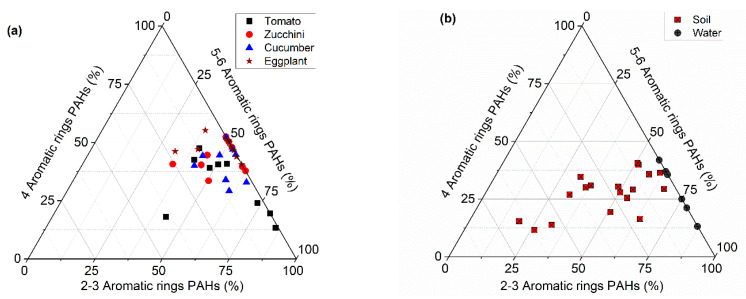
Ternary plot of PAH compositions in (**a**) vegetable samples and (**b**) water and soil samples.

The predominant types of PAHs detected in all types of the studied samples were the HMW-PAHs, as shown in Figure 4. However, the LMW-PAHs were found in higher concentrations in the soil, and it seems that these types are removed from the soil due to volatilization or degradation by micro-organisms far more easily than the more stable HMW-PAHs [59]. Although the log P of HMW-PAHs is higher than that of LMW-PAHs and their uptake into plants is stricter, it seems that their availability and stability in the soil contribute effectively to their transfer into the plants. In addition, the contamination of the vegetables through the air should be considered as the investigated area is close to traffic lines.

### 3.5. PAH Source Identification

Figuring out the relevant origins of PAHs in the plant samples and agricultural soil serves as a feasible way to establish a more vigorous and strategic course of action to restrict their emissions and diminish health risks. Different technique are able to be used for determining PAH sources, which involve molecular diagnostic ratios [49], PCA [30], and the positive matrix factorization (PMF) model [8].

#### 3.5.1. Determination of PAH Sources Using Diagnostic Ratios

**Figure 5:**
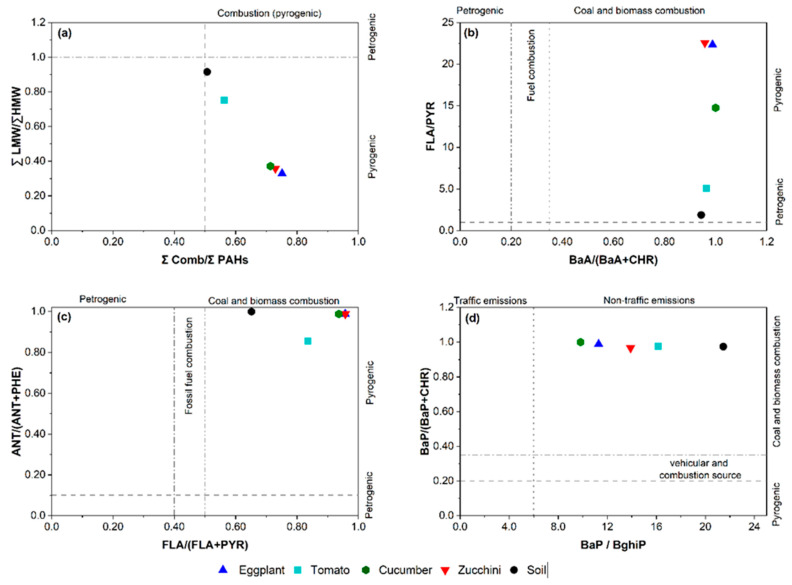
Cross-plots for the diagnostic ratios of: (**a**) LMW/HMW vs. ∑comb/∑16PAHs, (**b**) BaA/(BaA + CHR) vs. (FLA/PYR), (**c**) FLA/(FLA + PYR) vs. ANT/(ANT + PHE), and (**d**) BaP/BghiP vs. BaP/(BaP + CHR) in soil and vegetables of King Talal Dam (KTD) and south of Deir Alla (SDA) in the Jordan Valley, Jordan.

PAH diagnostic ratios have been widely applied to identify the main sources of PAHs (Table 2) [1,21,30]. In this research, the PAH isomer ratios such as BaP/(BaP + CHR), FLA/PYR, BaP/BghiP, BaA/(BaA + CHR), LMW/HMW, FLA/(FLA + PYR), and ANT/(ANT + PHE) were calculated, as shown in Table 1, to allocate sources of the detected PAHs in vegetable and soil samples, while Figure 5 displays the cross-plots of the approved diagnostic ratios of the vegetables and soil.

The LMW/HMW ratio values of the soil and vegetables were found to be lower than 1 (Figure 5), indicating a PAH contribution mainly from a pyrogenic origin. The BaA/(BaA + CHR) ratio values of the soil and vegetables were >0.35, indicating that the PAHs in the agricultural soil and vegetables were derived from wood and grass burning. However, the ratios of FLA/(FLA + PYR) > 0.5 and ANT/(ANT + PHE) > 0.1 were found in the soil and vegetables; this implied that the source of PAHs was pyrogenic, specifically, the burning of grass and wood.

In vegetables (cucumbers, eggplants, zucchini, and tomatoes) and agricultural soil, the BaP/BghiP ratio values exceeded >0.6, indicating traffic emissions might contribute to the occurrence of PAHs in the study area, especially mobile sources associated with the use of tractors and generators in agricultural activities. However, for cucumbers, eggplants, zucchini, tomatoes, and agricultural soil, the BaP/(BaP + CHR) ratio values were >0.35, indicating that the combustion of biomass might contribute to PAHs in the study area. As presented in Figure 5, the ratio FLA/PYR values of the soil and vegetables ranged from 1.87 to 22.56, which was higher than 1, indicating a pyrogenic source of PAHs.

Overall, based on the results of all of the diagnostic ratios above, the origins of the PAH contaminants in the soil and vegetables mainly originated from pyrogenic sources, traffic emission sources, and biomass burning.

#### 3.5.2. Source Identification by PCA

**Figure 6:**
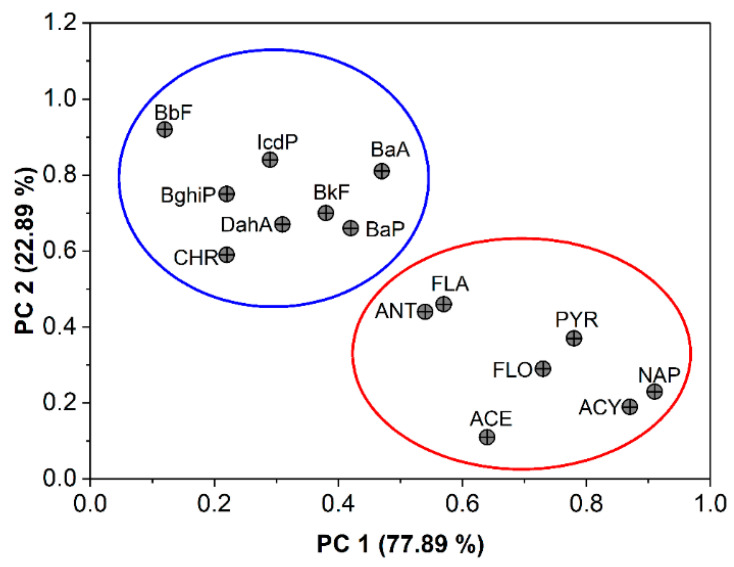
Principal component analysis (PCA) loading plot for 15 individual PAHs in soil.

In addition to the diagnostic ratios and in order to enhance the correctness of the source recognizing of PAHs in the vegetables and soil, PCA was applied with Varimax rotation. The results of the PCA in Figure 6 reveal that the two principal components (PC1 and PC2), with eigenvalues higher than 1, were extracted and accounted for 93.18% of the total variances. Each type of PAH source may provide an individual profile or signature. PC1 was responsible for 70.29% and was heavily weighted by NAP (0.91), ACY (0.87), ACE (0.64), FLO (0.73), ANT (0.54), FLA (0.57), and PYR (0.78). FLA, PYR, ANT, FLO, ACE, ACY, and NAP were recognized as residues of the PAH compounds originating from pyrogenic processes, with an example of wood and biomass (especially agricultural residue waste) burning [60,61]. Thus, PC1 represents the source of the open burning of agricultural waste. The burning of agricultural residue was ubiquitous in the studied area during a severe cold winter to save the crops against severe frost, resulting in significant amounts of PAHs being emitted in the atmosphere but mainly of the low molecular weight species [62].

However, PC2 explained 22.89% of the total variances and exhibited high loadings of BaA (0.81), CHR (0.59), BbF (0.92), BkF (0.70), BaP (0.66), IcdP (0.84), DahA (0.67), and BghiP (0.75). According to some authors [63], BghiP, DahA, BkF, IcdP, and BbF, species with a high molecular weight, were mainly from particles which are typically known to originate from vehicular emissions and fossil fuel combustion. The last group consists of BaP, CHR, and BaA, which were characteristic of diesel engine motorized vehicle sources [64]. Consequently, PC2 seems to represent a combination of diesel and traffic emission sources of PAHs, which might be a result of the use of agricultural machinery such as tractors and fuel portable generators in agricultural fields.

The PCA analysis has suggested biomass combustion and traffic emissions as the primary sources of PAH pollution.

### 3.6. PAH Bioconcentration in Vegetables

The bioconcentration factor (BCF) for PAHs is an indicator that displays the ratio of ∑16PAHs in vegetables to that in soils [2,65]. The soil–roots transfer of PAHs to vegetables is connected to the octanol/water partition coefficient (Kow) and their solubility in organic solvents [28]. The BCF is one of the crucial elements of human exposure to PAHs through the consumption of vegetables and/or food chains; therefore, it is essential to calculate the BCF (Table 3) of individual PAHs in various vegetables grown in the study region.

Tomatoes exhibited the highest BCF value (0.88) for ∑16 PAHs, followed by cucumbers (0.45), eggplants (0.45), and zucchini (0.43). In the group of the individual PAHs, the maximum BCF was observed for PHE (303.15) and BkF (5.38) in tomatoes, followed by PHE (5.35) in eggplants, PHE (4.98) in zucchini, and PHE (4.14) in cucumbers. It can be seen that the BCF value of high molecular weight PAHs (HMW-PAHs) was higher than the low molecular weight PAHs (LMW-PAHs). It indicates that HMW-PAHs have very great transportability. The BCF increases with the reduction in Kow and with the increase in PAH solubility in water [65]. BCFs are exactly associated to the availability of PAHs, which is influenced by soil organic matter; as a result, soil with high values of organic matter allows less bioaccumulation of PAHs in vegetables [66].

The PAH concentration in vegetables is controlled by plant characteristics, soil characteristics such as soil texture, pH, EC, organic matters, and properties, and the characteristics of the PAHs, the content of the PAHs in the growing media, and the transfer routes [20,66]. A BCF value > 1 indicates that the vegetable is an accumulator of the specific PAH under consideration. It is evident from Table 3, that the examined vegetables behave as accumulators for NAP, FLO, PHE, ANT, BaA, BbF, BkF, and BghiP. However, tomatoes are demonstrated to be hyperaccumulators of PHE (303.15), BkF (5.38), and BbF (2.03). Eggplants, zucchini, and cucumbers, in addition, behaved as hyperaccumulators of PHE as the BCFs values were 5.35, 4.98, and 4.14, respectively. These results indicate that the hyperaccumulating capacity of different vegetables towards PAHs differs with the physiology and growing circumstances of the vegetables.

A number of studies have stated that the uptake of leaves is the major cause of the accumulation of PAHs in vegetables [30,67]. The transfer of PAHs to vegetables happens by absorption in the gas phase via external cuticular lamellae or the deposition of particles on the leaves [1,28].

The BCF is high at a low PAH soil content and decreases at a higher PAH soil content [68]. A possible interpretation for this model is that vegetables have a slight sorption capacity for PAHs, which becomes saturated at a higher PAH soil content. Anyhow, the most logical explanation is that the absorption of PAHs into plants is from two different sources, specifically, soil and air. When the soil PAH content is very small, there still is background PAH pollution of the vegetable tissue arising from the air [68].

### 3.7. The Incremental Lifetime Cancer Risk from PAHs

Exposure to PAHs, one of the most common and concerning organic contaminants, is recognized to increase the cancer risk to human health [1]. Several studies, such as Aamir et al. [69], Han et al. [70], and Qi et al. [71] reported that exposure to PAHs could result in increased incremental lifetime cancer risk (ILCR) in humans. Due to the significance of PAH bioaccumulation in human bodies via vegetable intake, we computed the ILCR (Figure 6) of the target population in their lifetimes from PAH exposure through the consumption of vegetables. The ILCR was demonstrated as the number of expected cases in excess of one in a million (10^−6^).

**Figure 7:**
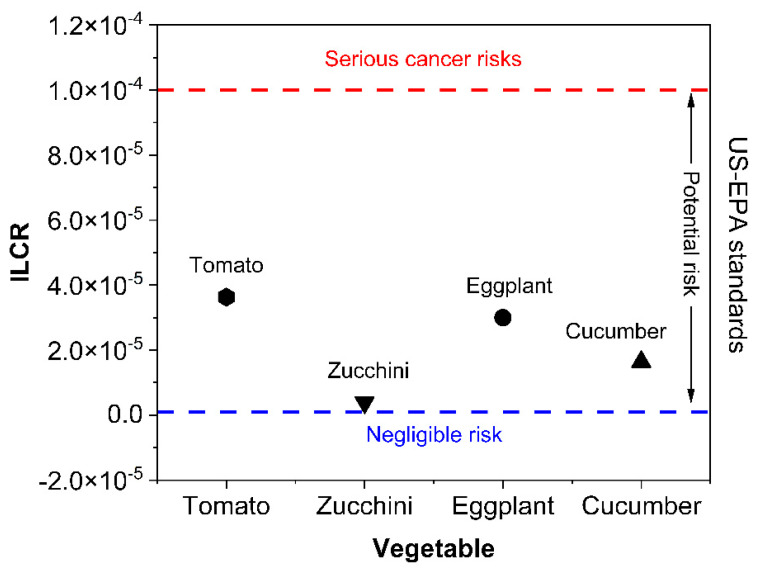
ILCR for PAHs in the different vegetables based on US-EPA standards.

According to the US-EPA standard, the ILCRs values <10^−6^, 10^−6^–10^−4^, and >10^−4^ were taken to denote negligible risk, potential risk, and serious high cancer risks, respectively [72,73]. In this research, the ILCR for PAH dietary exposure was 3.63 × 10^−5^, 3.86 × 10^−6^, 3 × 10^−5^, and 1.64 × 10^−5^ for tomatoes, zucchini, eggplants, and cucumbers, respectively. The results of this research proposed a serious potential cancer risk related to the tomatoes, zucchini, eggplants, and cucumbers (Figure 7) from exposure to lifetime eating of the examined vegetable items. It was noted that the cumulative ILCR for PAH dietary exposure to the four vegetables (tomatoes, zucchini, eggplants, and cucumbers) evaluated in this study was 8.68 x 10−5. Consumption of the chosen vegetables in the investigated area acts to cause a potential risk of cancer with reference to the PAHs. Societal risk was computed by multiplying the ILCR with Jordan’s current inhabitants (10 million), and it was found that 86 extra cases of cancer are probably because of lifetime consumption exposure to PAHs at their identified concentrations.

Exposure to PAHs in the long-term, promoted by dietary exposure to locally cultivated vegetables, could result in PAHs accumulating in human tissues as PAHs are lipophilic and have high biological half-lives [74]. Thus, it is serious and important to take suitable action to control the potential health risks for Jordanian citizens from dietary exposure to PAHs.

## 4. Conclusions

This work focused on the quantitation of 16 PAHs in different types of vegetables, farmland soil, and irrigation water in the Jordan Valley. The farm soil showed the higher mean concentration of the total PAHs among all of the studied samples, while the water samples were the lowest. Among all of the vegetable types, tomatoes contained the highest levels of total PAHs, while zucchini showed the lowest level. The residue analysis showed that the concentration of the PAHs depends not only on the characteristics of the individual PAH compound but even more on the vegetable types. As all the vegetables were grown up under the same environmental conditions and irrigated from the same water, the PCA results reveal that the PAHs in the agriculture soil samples originated mainly from biomass burning and vehicular emissions. The estimated values of the cancer risk indices for all of the vegetable species clearly indicated that the consumption of the grown vegetables in the Jordan Valley could pose a potential cancer risk to human health. As a result, more attention from the local authorities and policymakers is required. Further, various risk management strategies and tools can be applied, such as the regular estimation of the contamination levels in different vegetable samples and planning awareness campaigns such as seminars and workshops to teach farmers about the risks of PAHs, the main routes they can contaminate their vegetables, and how to prevent or reduce the sources of these pollutants as well as use sustainable agricultural practices through avoiding biomass combustion.

## Figures and Tables

**Table 1:** Mean (µg kg^−1^ for plants and soil and µg kg^−1^ for water), standard deviation, and isomer ratio of the PAHs in the studied samples.

Target Compound	Abb.	Mean Eggplant *n* = 10	SD	% Det	Mean Tomato *n* = 9	SD	% Det	Mean Zucchini. *n* = 9	SD	% Det	Mean Cucumber *n* = 9	SD	% Det	Mean Soil *n* = 18	SD	% Det	Mean Water *n* = 7	SD	% Det
Naphthalene	NAP	0.094	0.083	20	0.287	0.243	78	1.453	-	11	0.690	0.672	22	2.276	0.894	100	BQL	-	
Acenaphthylene	ACY	0.586	0.133	20	0.335	0.187	22	BQL	-	--	BQL	-	--	1.879	1.295	39	BQL	-	--
Acenaphthene	ACE	0.408	0.461	20	1.570	0.507	44	BQL	-	--	0.708	0.359	33	4.350	1.565	28	BQL	-	
Fluorene	FLO	0.111	-	10	BQL	-	--	BQL	-	--	0.472	-	11	0.404	0.556	50	BQL	-	--
Phenanthrene	PHE	0.018	0.009	30	1.040	1.395	22	0.017	-	44	0.014	0.001	22	0.007	-	6	BQL	-	--
Anthracene	ANT	1.533	-	10	6.119	-	11	1.335	0.681	44	1.164	-	11	4.869	3.169	33	BQL	-	--
Fluoranthene	FLA	0.812	0.227	100	0.986	0.470	89	0.838	0.339	100	0.656	0.170	89	0.918	0.546	100	0.019	0.005	43
Pyrene	PYR	0.036	0.013	70	0.194	0.231	100	0.037	0.036	100	0.045	0.0170	89	0.491	0.273	100	0.002	0.001	43
Benzo[a]anthracene	BaA	3.236	1.014	100	3.718	2.173	89	2.641	0.629	100	2.807	0.500	100	3.059	1.072	100	0.066	0.016	71
Chrysene	CHR	0.039	0.035	50	0.137	0.133	56	0.116	-	11	BQL	-	--	0.182	0.120	94	0.002	0.001	43
Benzo[b]fluoranthene	BbF	0.516	0.105	90	0.776	0.517	100	0.342	0.095	100	0.441	0.261	100	2.330	0.162	94	0.013	0.003	100
Benzo[k]fluoranthene	BkF	0.017	0.009	90	0.280	0.533	44	0.013	0.009	33	0.009	0.003	44	0.071	0.096	22	0.001	0.001	57
Benzo[a]pyrene	BaP	3.234	1.268	100	5.436	2.127	100	3.333	1.429	100	3.471	1.077	100	6.776	2.780	100	0.096	0.039	100
Indeno[1.2.3.4.cd]pyrene	IcdP	0.179	0.063	80	0.384	0.272	78	0.207	0.094	100	0.251	0.203	100	0.414	0.224	67	0.010	0.004	43
Dibenzo[a.h]anthracene	DahA	0.014	0.020	20	0.178	0.194	33	0.077	0.023	33	0.176	0.146	44	0.382	0.477	39	0.002	-	14
Benzo[g.h.i]perylene	BghiP	0.286	0.142	90	0.337	0.337	100	0.240	0.082	100	0.353	0.336	100	0.316	0.375	94	0.009	0.005	100
∑PAHs		11.120			21.774			10.649			11.256			28.724			0.218		
∑COMB		8.355			12.247			7.767			8.032			14.557			-		
∑COMB/∑PAHs		0.751			0.562			0.729			0.714			0.507			-		
∑LMW PAHs		2.751			9.350			2.805			3.048			13.785			-		
∑HMW PAHs		8.369			12.424			7.844			8.208			14.939			-		
∑LMW/∑HMW		0.329			0.753			0.358			0.371			0.923			-		
BaP/BghiP		11.292			16.149			13.897			9.828			21.464			-		
BaP/(BaP + CHY)		0.988			0.975			0.966			1.000			0.974			-		
BaA/(BaA + CHR)		0.988			0.965			0.958			1.000			0.944			-		
FLA/PYR		22.332			5.071			22.556			14.749			1.870			-		
ANT/(ANT + PHE)		0.988			0.855			0.987			0.988			0.999			-		
FL/(FL + PYR)		0.957			0.835			0.958			0.937			0.652			-		

BQL: below quantitation limit; % Det: percentage of the detected samples; ∑COMB-(FLA, PYR, BaA, CHR, BkF, BbF, BaP, IcdP and BghiP).

**Table 2:** Guidelines for PAH diagnostic ratios considered for identifying sources of PAHs in vegetables and soil [1,2,15,24].

Diagnostic Ratio	Value	Source Apportionment
ANT/(ANT + PHE)	<0.1	petroleum (petrogenic source)
	>0.1	combustion (pyrogenic sources)
FLA/(FLA + PYR)	<0.4	petrogenic
	0.4–0.5	liquid fossil fuel burning
	>0.5	coal, grass, and wood burning
BaA/(BaA + CHR)	<0.2	petroleum (petrogenic source)
	0.2–0.35	fuel combustion (vehicular emissions)
	>0.35	coal, grass, and wood burning
Bap/(Bap + CHR)	<0.20	petrogenic origin
	0.2 −0.35	vehicular and combustion source
	>0.35	coal, wood, and grass burning source
BaP/BghiP	>0.6	traffic emissions
	<0.6	nontraffic emissions
FLA/PYR	<1	petrogenic source
	>1	pyrogenic source
LMW/HMW	<1.0	pyrogenic sources such as coal, grass, and burning of wood
	>1.0	petrogenic sources, such as fuel or one refined petroleum product
∑comb/∑16PAHs	~1	combustion

ΣLMW = sum of LMW-PAHs (2–3 rings); ΣHMW = sum of the HMW-PAHs (4–6 rings); ∑COMB-(FLA, PYR, BaA, CHR, BkF, BbF, BaP, IcdP and BghiP).

**Table 3:** Bioconcentration factor (BCF) of vegetables collected from King Talal Dam (KTD) and south of Deir Alla (SDA) in the Jordan Valley.

16 PAHs	# of Rings	BCF Cucumber	BCF Eggplant	BCF Zucchini	BCF Tomato
NAP	2	0.54	0.07	1.14	0.22
ACY	3	ND	0.31	ND	0.18
ACE	3	0.19	0.11	ND	0.43
FLO	3	1.79	0.42	ND	ND
PHE	3	4.14	5.35	4.98	303.15
ANT	3	0.24	0.32	0.28	1.26
FLA	4	0.71	0.88	0.91	1.07
PYR	4	0.09	0.07	0.08	0.40
BaA	4	0.92	1.06	0.86	1.22
CHR	4	ND	0.22	0.64	0.75
BbF	5	1.16	1.35	0.90	2.03
BkF	5	0.17	0.32	0.25	5.38
BaP	5	0.51	0.48	0.49	0.80
IcdP	6	0.65	0.46	0.54	0.99
DahA	6	0.55	0.05	0.24	0.56
BghiP	6	1.17	0.95	0.79	1.11
∑16 PAHs		0.45	0.45	0.43	0.88
∑LMW-PAHs		0.26	0.23	0.24	0.79
∑HMW-PAHs		0.64	0.65	0.61	0.97

## Data Availability

The data presented in this study are available on request from the corresponding author.
